# Pneumococcal vaccination responses in adults with subnormal IgG subclass concentrations

**DOI:** 10.1186/s12865-019-0310-3

**Published:** 2019-08-20

**Authors:** Antony R. Parker, Markus Skold, Stephen Harding, J. Clayborn Barton, Luigi F. Bertoli, James C. Barton

**Affiliations:** 1 The Binding Site Group Limited, 8 Calthorpe Road, Birmingham, B15 1QT UK; 2 Southern Iron Disorders Center, Birmingham, AL USA; 3 Department of Medicine, Brookwood Medical Center, Birmingham, AL USA; 4 Department of Medicine, University of Alabama at Birmingham, Birmingham, AL USA

**Keywords:** IgG subclasses, Vaccine response, Pneumococcal, Pneumovax®23, Primary immunodeficiency

## Abstract

**Background:**

We sought to compare Pneumovax®23 responses in adults with subnormal IgG subclass concentrations. We studied adults with normal total IgG, frequent/severe respiratory infection, and subnormal IgG1, IgG3, or IgG1 + IgG3 before and after Pneumovax®23. We defined response as serotype-specific IgG > 1.3 μg/mL and aggregate response as IgG > 1.3 μg/mL for ≥70% of all serotypes tested. We compared patients with and without serotype-specific responses and performed logistic regression on aggregate responses using: age; male sex; body mass index; autoimmune condition(s); atopy; other allergies; subnormal IgGSc immunophenotypes; IgA; and IgM.

**Results:**

There were 59 patients (mean age 44 ± 13 (SD) years; 83.1% women). Median days between pre- and post-Pneumovax®23 testing was 33 (range 19–158). The median post-vaccination summated concentration of serotype-specific IgG was higher in patients with subnormal IgG1 than subnormal IgG3 (responders and non-responders). All subnormal IgG1 + IgG3 non-responders responded to serotypes 8, 9 and 26, unlike other non-responders. Subnormal IgG3 responders had lower responses to serotypes 1, 4, 12, 23, 26, and 51. Subnormal IgG3 non-responders had higher responses to serotypes 1, 3, 8, 9, 12, 14, 19, 51, and 56. Response rates decreased with increasing age. Aggregate responders were: subnormal IgG1, 54%; IgG3, 46%; and IgG1 + IgG3, 46%. Regression on aggregate response revealed lower response with male sex (odds ratio 0.09 [95% CI 0.01, 0.77]) and atopy (0.17 [0.03, 0.83]).

**Conclusions:**

Serotype-specific IgG responses to Pneumovax®23 were greater in patients with subnormal IgG1 than subnormal IgG3. Male sex and atopy were associated with lower aggregate responses.

## Background

Subnormal IgG subclass (IgGSc) concentrations in some adults represent a primary immunodeficiency disorder(s) characterized by frequent or severe respiratory tract infections, suboptimal IgG responses to pneumococcal polysaccharides, and increased prevalence of autoimmune disorders and atopy [1–3]. The subnormal serum concentrations are mainly present in any one or more of three main subclasses IgG1, IgG2, or IgG3 [4, 5]. Molecules of the four respective IgG subclasses are characterized by distinct induction antigens or allergens, antigen binding, immune complex formation, complement activation, half-life, and other properties. Accordingly, patients with different subnormal IgGSc immunophenotypes have dissimilar predisposition to infection [6].

IgG1 production is induced by exposure to soluble and membrane protein antigens and allergens. Some individuals with subnormal IgG1 have frequent or severe infections, especially of the respiratory tract [7, 8]. Antibody responses to bacterial capsular polysaccharide antigens are associated with low serum IgG2 [6, 9, 10]. Some persons with subnormal IgG2 have increased risk of respiratory tract infections due to encapsulated bacteria, although others are apparently healthy [11, 12]. Some persons with subnormal IgG3 also have subnormal concentrations of other subclasses, especially IgG1 [13, 14]. Some allergens and parasites elicit IgG4 responses [15, 16], but the clinical significance of subnormal IgG4, if any, is unclear.

Some patients with subnormal IgGSc respond to certain vaccines and others do not [8, 17–19]. In a study of 17 patients with subnormal IgGSc, 14 patients who responded to 23-valent pneumococcal polysaccharide vaccination (PPSV23) had no further progression of respiratory tract infections, whereas three PPSV23 non-responders required antibiotic treatment [20]. van Kessel and colleagues reported that following vaccination with PPSV23, non-responders with subnormal IgG1 to the measured 14 pneumococcal polysaccharide serotypes (PPS), had increased risk of infection with *Streptococcus pneumoniae,* and a greater proportion had received treatment with corticosteroids [21]. Because IgGSc levels and vaccination response are independent but overlapping markers of B-lymphocyte and plasma cell function, it is plausible that the combination of a lack of vaccination response and subnormal IgGSc increases risk of corresponding infection(s).

In this report, we describe a retrospective analysis of characteristics of 59 adults with subnormal IgGSc concentrations but normal IgG. We report whether they did or did not respond to PPSV23 and their concentrations of pre- and post-PPSV23 serotype-specific IgG. We discuss the relationships between subnormal IgGSc immunophenotypes and other attributes of the present patients with specific and aggregate IgG responses to PPSV23 and the pertinence of the present results to infection susceptibility and prevention in patients with subnormal IgGSc.

## Methods

### Subject selection

Performance of this work was reviewed by the Institutional Review Board of Brookwood Medical Center, Alabama. Obtaining informed consent was not required because this study involved evaluation of observations obtained in routine medical care. We studied the records of unrelated non-Hispanic white adults (≥18 years of age) referred to a single practice in a large suburban medical center that evaluates and treats many adult patients with primary immunodeficiency. All patients presented with frequent or severe bacterial infections of the upper or lower respiratory tract and were diagnosed to have IgG subclass deficiency [22]. By selection, all of the present patients had normal total serum IgG. Autoimmune conditions, atopy, and other allergy manifestations were defined as previously described [23]. The present patients were evaluated during the interval 2012–2018.

### Other conditions

Body mass index was computed as kg/m^2^. We classified diabetes according to the criteria of the American Diabetes Association [24].

### Polyvalent pneumococcal polysaccharide vaccination

*S. pneumoniae* serotype-specific IgG concentrations were measured before and after PPSV23 (Pneumovax®23; Merck & Co., Inc., Kenilworth NJ, USA). The median interval between pre- and post-PPSV23 testing was 33 days (range 19–158 days).

On 30 December 2011, PCV13 (Prevnar13®, Wyeth Pharmaceuticals, Inc., Philadelphia, PA, USA) was approved for use in the US among adults aged ≥50 years to prevent pneumonia and invasive disease caused by *S. pneumoniae* serotypes contained in the vaccine [25]. On 13 August 2014, the routine use of PCV13 among adults aged ≥65 years was recommended in series with PPSV23, the vaccine then recommended for adults aged ≥65 years [25]. In late 2018, the US Centers for Disease Control and Prevention recommended pneumococcal vaccination (both PPSV23 and PCV13) for adults ages 19–64 years who have certain medical conditions or who smoke, including those with congenital or acquired immunodeficiency. The US Centers for Disease Control and Prevention now recommends pneumococcal vaccination for all adults 65 years or older (both PPSV23 and PCV13) [26]. Although none of the present adults reported that they had received PCV13, the possibility that some of them received PCV13 as children cannot be excluded.

### Laboratory methods

All testing was performed before IgG replacement therapy was initiated. Serum Ig concentrations were measured using standard methods at a single reference laboratory (Laboratory Corporation of America, Burlington NC, USA). We defined mean ± 2 SD as reference ranges for all Ig concentrations. Ig reference ranges employed were: IgG 7.0–16.0 g/L (700–1600 mg/dL); IgG1 4.2–12.9 g/L (422–1292 mg/dL); IgG2 1.2–7.5 g/L (117–747 mg/dL); IgG3 0.4–1.3 g/L (41–129 mg/dL); IgG4 0–2.9 g/L (1–291 mg/dL); IgA 700–4000 mg/L (70–400 mg/dL); and IgM 400–2300 mg/L (40–230 mg/dL). Subnormal Ig concentrations were defined as those below the corresponding reference limits.

Pre- and post-PPSV23 serotype-specific IgG antibodies were measured by clinical laboratories (Laboratory Corporation of America, Burlington NC, USA and ViraCor-IBT, Lee’s Summit MO, USA) and included *S. pneumoniae* serotypes 1, 3, 4, 8, 9. 12, 14, 19, 23, 26, 51, 56, 57, and 68. Diluents for patient samples contained C-polysaccharide and polysaccharide type 22. The sums of post-PPSV23 serotype-specific IgG concentration in test panels were defined as summated concentrations.

### Response to PPSV23

We defined protective serotype-specific IgG levels as > 1.3 μg/mL. We defined responders as patients who achieved protective levels of serotype-specific IgG post-PPSV23. We defined aggregate PPSV23 response as serotype-specific IgG > 1.3 μg/mL for ≥70% of serotypes tested post-PPSV23.

### Statistics

The analytic data set consisted of complete observations on 59 patients with subnormal IgGSc and total serum IgG > 700 mg/dL. For some analyses, we separated patient data into three groups: 1) subnormal IgG1; 2) subnormal IgG3; and 3) subnormal IgG1 + IgG3. Descriptive data are displayed as enumerations, percentages, mean ± 1 SD, or mean 95% confidence intervals (CI). Age and BMI data were normally distributed and were compared using Student’s t-test (two-tailed*).* Proportions were compared using Pearson’s χ^2^ test or Fisher’s exact test, as appropriate. We computed odds ratios (OR) 95% CI for some observations. Means of continuous variables among three patient subgroups were compared using one-way analysis of variance (ANOVA) or Kruskal-Wallis one-way ANOVA, as appropriate. Median concentrations were compared using Mann-Whitney U Test and Spearman’s rank test to assess the association between pneumococcal summation and coverage. Dichotomous variables in frequency analyses were: male; diabetes; autoimmune condition(s); atopy (allergic asthma, allergic rhinitis, or allergic dermatitis/eczema); and other allergy (urticaria, angioedema, or anaphylaxis). Total clinical variables were calculated from these dichotomous variables.

We compared the general characteristics of patients who did or did not respond to pneumococcal polysaccharides using univariate analyses. We also performed logistic multivariable regressions on aggregate IgG response to pneumococcal polysaccharides to identify significant associations using these independent variables: age at diagnosis; male sex; body mass index (BMI); autoimmune condition(s); atopy; other allergies; subnormal IgG1; subnormal IgG3; subnormal IgG1 + IgG3; and serum levels of IgA and IgM. We computed OR of independent variables with significant associations. Dichotomous variables in frequency analyses were: male; diabetes; autoimmune condition(s); atopy (allergic asthma, allergic rhinitis, or allergic dermatitis/eczema); and other allergy (urticaria, angioedema, or anaphylaxis). Total clinical variables were calculated from these dichotomous variables. Analyses were performed with GraphPad Prism v. 5.04 (GraphPad Software, Inc., La Jolla, CA, USA), Excel 2000® (Microsoft Corp., Redmond, WA, USA), and GB-Stat® (v. 10.0, 2003, Dynamic Microsystems, Inc., Silver Spring, MD, USA). We defined values of *p* < 0.05 to be significant. Bonferroni corrections were applied to control the type I error rate at 0.05 for multiple univariate comparisons.

## Results

### General characteristics of patients with subnormal IgGSc

The proportions of deficiencies in 59 patients with subnormal IgGSc were: subnormal IgG1 22.0% (*n* = 13); subnormal IgG3 55.9% (*n* = 33); and subnormal IgG1 + IgG3 22.0% (*n* = 13) (Table 1).

**Table 1:** Characteristics of 59 index patients with subnormal IgGSc and normal total IgG^a^

Characteristic	Subnormal IgG1	Subnormal IgG3	Subnormal IgG1+ IgG3	All patients
n	13	33	13	59
Mean age, years ± SD	34 ± 13	45 ± 12	48 ± 13	44 ± 13
Men, % (n)	23.1 (3)	6.1 (2)	38.5 (5)	16.9 (10)
Diabetes, % (n)	0	6.1 (2)	15.1 (2)	6.8 (4)
Mean BMI, kg/m^2^ ± SD	27.3 ± 7.8	26.6 ± 5.7	30.8 ± 12.8	28.1 ± 8.2
Autoimmune condition(s), % (n)	23.1 (3)	42.4 (14)	30.8 (5)	37.3 (22)
Atopy^b^, % (n)	23.1 (3)	36.4 (12)	15.4 (2)	28.8 (17)
Other allergy^c^, % (n)	53.8 (7)	51.5 (17)	46.2 (6)	50.8 (30)
Mean IgA, mg/dL ± SD	154 ± 74	206 ± 106	196 ± 91	200 ± 95
Mean IgM, mg/dL ± SD	263 ± 86	120 ± 99	104 ± 75	117 ± 90
Median days between pre-, post-PPSV23 tests (range)	32 (27–47)	35 (19–129)	32 (19–158)	33 (19–158)

^a^*Abbreviations*: *Ig* Immunoglobulin, *BMI* Body mass index, *PPSV23* Polyvalent pneumococcal polysaccharide vaccination. IgG subclass levels were measured before IgG replacement therapy. Mean ± 2 SD was defined as reference ranges for all Ig measurements. Ig reference ranges are: total IgG 7.0–16.0 g/L (700–1600 mg/dL); IgG1 4.2–12.9 g/L (422–1292 mg/dL); IgG2 1.2–7.5 g/L (117–747 mg/dL); IgG3 0.4–1.3 g/L (41–129 mg/dL); IgG4 0–2.9 g/L (1–291 mg/dL); IgA 700–4000 mg/L (70–400 mg/dL); IgM 400–2300 mg/L (40–230 mg/dL). Subnormal Ig concentrations were defined as those below the corresponding reference limits

^b^Allergic asthma, allergic rhinitis, or allergic dermatitis/eczema

^c^Urticaria, angioedema, or anaphylaxis

Two patients (3.7%) had subnormal IgA. Three patients (5.1%) had subnormal IgM. χ^2^ analysis of the proportions of men in the three subnormal IgGSc groups rejected the null hypothesis (*p* = 0.03). This was due to the lower proportion of men with subnormal IgG3 than subnormal IgG1 + IgG3 (6.1% vs. 38.5%, respectively; *p* = 0.014) (Table 1). Proportions of patients with diabetes, autoimmune condition(s), atopy, and other allergy did not differ significantly among the three groups. One-way ANOVA revealed no significant differences of age, BMI, serum IgA, serum IgM or days between pre- and post-PPSV23 testing among the three groups (Table 1).

In two of the three subnormal IgGSc groups, there was a significant positive Spearman rank correlation of total levels of pneumococcal serotype-specific IgG and percentage serotype coverage per patient (subnormal IgG1, rho = 0.66, *p* = 0.02; subnormal IgG3, rho = 0.8, *p* < 0.0001; and subnormal IgG1 + IgG3, rho = 0.52, *p* = 0.07).

### Serotype-specific IgG

**Fig. 1:**
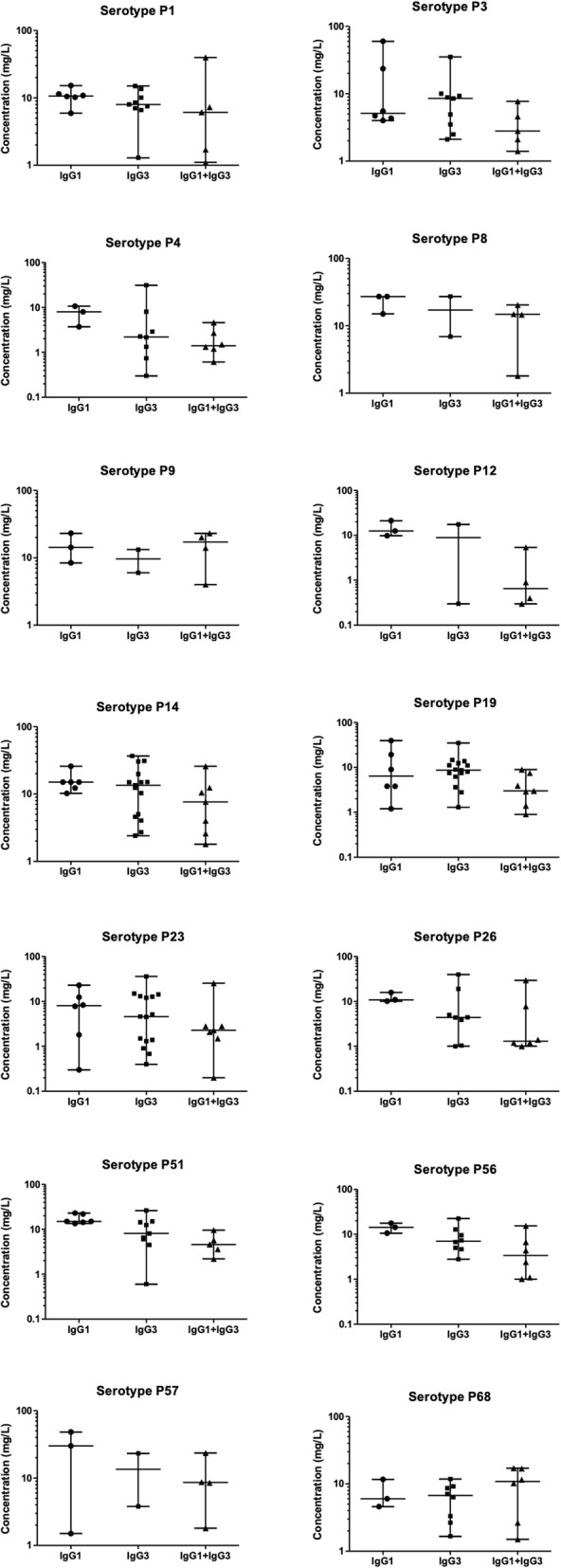
*S. pneumoniae* serotype-specific IgG concentrations in subnormal IgGSc PPSV23 responders. Lines represent minimum, median, and maximum concentrations

**Fig. 2:**
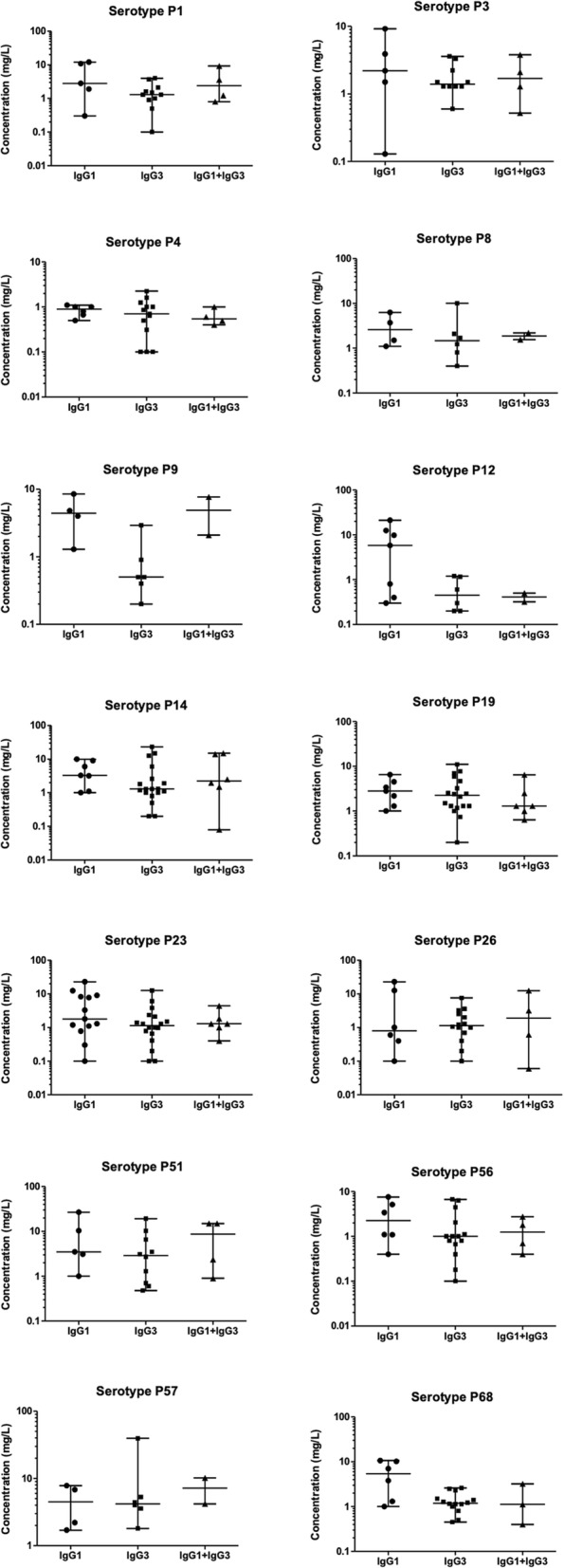
*S. pneumoniae* serotype-specific IgG concentrations in subnormal IgGSc PPSV23 non-responders. Lines represent minimum, median, and maximum concentrations

Serum concentrations of anti-pneumococcal serotype specific IgG in the different subnormal IgGSc groups are displayed in Figs. 1 and 2. In PPSV23 responders, the trend in anti-pneumococcal IgG was subnormal IgG1 > subnormal IgG3 > subnormal IgG1 + IgG3. For serotypes 3, 19 and 68, the median concentration of anti-pneumococcal IgG was greater in patients with subnormal IgG3 than in patients with subnormal IgG1 and for serotypes 9 and 68 was higher in patients with subnormal IgG1 + IgG3 than patients with subnormal IgG3. In PPSV23 non-responders, the trend of serum levels of anti-pneumococcal antibodies was subnormal IgG1 > subnormal IgG1 + IgG3 > subnormal IgG3. For serotypes 26 and 57, the concentrations of anti-pneumococcal IgG were higher in patients with subnormal IgG3 than in patients with subnormal IgG1.

We investigated the percentage response and non- response to each serotype tested in responders and non-responders across the three subnormal IgGSc immunophenotype groups (Table 2).

**Table 2:** Non-responders to pneumococcal serotypes in 59 index patients with subnormal IgGSc and normal total IgG^a^

*S. pneumoniae* serotype	1	3	4	8	9	12	14	19	23	26	51	56	57	58
Subnormal IgG1, % NR	20	20	100	25	25	75	29	29	71	66	20	50	0	17
Subnormal IgG1, % R	0	0	0	0	0	0	0	17	17	0	0	0	0	0
Subnormal IgG3, % NR	55	50	85	50	85	100	56	40	72	64	40	71	0	64
Subnormal IgG3, % R	11	0	25	0	0	50	0	7	27	25	13	0	0	0
Subnormal IgG1 + IgG3, % NR	50	50	100	0	0	100	17	66	66	0	25	50	0	66
Subnormal IgG1 + IgG3, % R	20	0	33	0	0	75	0	14	14	0	0	33	0	0

^a^*Abbreviations*: *IgGSc* IgG subclass(es), *NR* Non-responders, *R* Responders. We defined responders as patients who achieved protective levels of serotype-specific IgG post- polyvalent pneumococcal polysaccharide vaccination

One responder in each immunophenotype group had 100% response to serotypes 3, 8, 9, 14, 26 and 68. Non-responders with subnormal IgG1 + IgG3 had 100% response to serotypes 8, 9 and 26, unlike non-responders with subnormal IgG1 or subnormal IgG3. Among patients with subnormal IgG3, non-responders had less response to serotypes 1, 3, 8, 9, 12, 14, 19, 51, 56 and the responders had less response to serotypes 1, 4, 12, 23, 26 and 51 (Table 2).

### Summated serotype-specific IgG

**Fig. 3:**
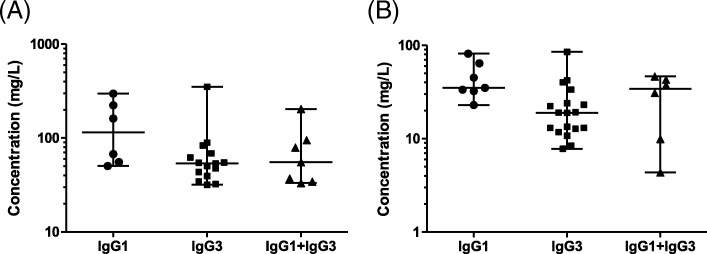
Summation of *S. pneumoniae* serotype-specific IgG antibodies. We measured the concentration of all serotype-specific antibodies per patient in subnormal IgGSc groups in **a** PPSV23 responders (IgG1, *n* = 6; IgG3, *n* = 15; and IgG1 + IgG3, *n* = 7) and **b** PPSV23 non-responders (IgG1, *n* = 7; IgG3, *n* = 18; and IgG1 + IgG3, *n* = 6)

The summated concentrations of serotype-specific IgG antibodies differed across the subnormal IgGSc groups (Fig. 3). In responders, we observed: subnormal IgG1, median 115 μg/mL (range 50–297); subnormal IgG3, median 54 μg/mL (range 32–351); and subnormal IgG1 + IgG3, median 55 μg/mL (range 33–204). In non-responders, we observed: subnormal IgG1, median 35 μg/mL (range 23–81); subnormal IgG3, median 19 μg/mL (range 8–85); and subnormal IgG1 + IgG3, median 34 μg/mL (range 4–47). For all comparisons, *p* = 0.04 (Fig. 3). The median summated concentration of serotype-specific IgG antibodies was significantly higher in subnormal IgG1 non-responders than subnormal IgG3 non-responders (*p* = 0.01).

**Fig. 4:**
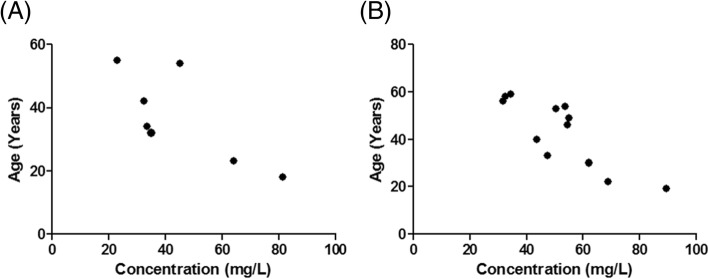
Influence of age on *S. pneumoniae* serotype-specific IgG antibodies. Summation of total *S. pneumoniae* serotype antibodies was positively associated with age in **a** subnormal IgG1 non-responders and **b** subnormal IgG3 responders

The summated concentrations of serotype-specific IgG decreased with increasing age, and were significant in non-responders with subnormal IgG1 (rho = − 0.79, *p* = 0.048) and subnormal IgG3 (rho = − 0.71, *p* = 0.007) (Fig. 4), but were not significantly associated with BMI or serum levels of IgG1, IgG3, IgG4, IgA, or IgM (data not shown).

### Clinical variables and PPSV23 response

The prevalence of clinical variables (total number of variables and atopy) was higher in PPSV23 non-responders than responders for all three subnormal IgGSc immunophenotype groups and in patients with subnormal IgG3 than in patients with subnormal IgG1 and subnormal IgG1 + IgG3. The prevalence of total clinical variables and atopy per person was higher in patients with subnormal IgG3 and PPSV23 non-responsiveness than those who did respond (1.3 vs. 0.4, *p* = 0.009 and 1.6 vs. 0.1, *p* = 0.003, respectively) (Table 3).

**Table 3:** Pneumococcal serotype-specific IgG responses and clinical variables in 59 adults with selective subnormal IgG3^a^

*S. pneumoniae* Serotype	Variable	Frequency of dichotomous variables		*P* value
		Responders^b^	Non-responders	
3	Atopy	0.4	1.3	0.009
4	Atopy	0	1.6	0.007
14	Atopy	0.1	1.3	0.005
56	Atopy	0	1.6	0.007
68	Atopy	0	1.6	0.007
1	Total variables	0.2	1.1	0.012
3	Total variables	0.1	1.6	0.003
14	Total variables	0.2	1.3	0.01

^a^Variables were defined as: male; diabetes; autoimmune condition(s); atopy (allergic asthma, allergic rhinitis, or allergic dermatitis/eczema); and other allergy (urticaria, angioedema, or anaphylaxis). Frequency was calculated as the mean number of each variable or total variables per patient in polyvalent pneumococcal polysaccharide vaccination responders and non-responders

^b^We defined responders as patients who achieved protective levels of serotype-specific IgG post- polyvalent pneumococcal polysaccharide vaccination. We defined protective serotype-specific IgG levels as > 1.3 μg/mL

Within groups of PPSV23 non-responders, the frequency of total clinical variables per person was higher in patients with subnormal IgG3 than in patients with subnormal IgG1 (1.3 vs. 0.4, respectively; *p* = 0.04). The median weight of PPSV23 non-responders with subnormal IgG1 was greater than that of PPSV23 non-responders with subnormal IgG3 (171 pounds (range 148–322) vs. 136 pounds (range 104–255), respectively; *p* = 0.03).

### Clinical variables and serotype-specific IgG responses

There was a significant difference between the prevalence of atopy and total clinical variables in non-responders compared with the corresponding data of responders with subnormal IgG3, but there were no significant differences between responders and non-responders in either the subnormal IgG1 or subnormal IgG1 + IgG3 groups (data not shown). In patients with subnormal IgG3 who were non-responders, atopy was associated with higher concentrations of IgG specific for antigens 3, 4, 14, 56 and 68, and the total number of clinical variables was associated with higher concentrations of IgG specific for serotypes 1, 3, and 14 (Table 3). There was a strong positive association between low concentration of 1, 3, 14, and 51 serotype-specific IgG in subnormal IgG3 PPSV23 responders and an association with male gender (Table 4*).* In PPSV23 non-responders, lower concentrations of some serotype-specific IgG were associated with a higher prevalence of: atopy (serotypes 4, 14, 56 and 68); autoimmune condition(s) (serotypes 3 and 51); other allergies (serotypes 3 and 51) and total clinical variables (serotype 14) (Table 4).

**Table 4:** Frequencies of variables that differed significantly between different IgG subclass deficiency subgroups^a^

*S. pneumoniae* Serotype	Dichotomous variable^b^	Frequency of variable			*P* value
		IgG1	IgG3	IgG1 + IgG3	
Responders^b^					
1	Male	0.8	0	0.7	0.006
3	Male	0.8	0	0.7	0.006
14	Male	0	0	0.5	0.01
51	Male	0.8	0	0.7	0.006
Non-responders					
4	Atopy	0.06	1.6	0	0.02
14	Atopy	0.17	1.3	0.2	0.04
56	Atopy	0.06	1.6	0	0.02
68	Atopy	0.06	1.6	0	0.02
3	Autoimmune condition(s)	0	1.6	0.2	0.02
51	Autoimmune condition(s)	0	1.6	0.2	0.02
19	Male	0.8	0.06	1	0.03
23	Male	0.8	0.06	1	0.03
3	Other allergy	0.4	1.3	0.4	0.04
51	Other allergy	0.4	1.3	0.4	0.04
14	Total variables	0.4	1.3	0.4	0.03

^a^Variables were defined as: male; diabetes; autoimmune condition(s); atopy (allergic asthma, allergic rhinitis, or allergic dermatitis/eczema); and other allergy (urticaria, angioedema, or anaphylaxis). Frequency was calculated as the mean number of each variable or total variables per patient in polyvalent pneumococcal polysaccharide vaccination responders and non-responders. ^b^We defined responders as patients who achieved protective levels of serotype-specific IgG post-polyvalent pneumococcal polysaccharide vaccination. We defined protective serotype-specific IgG levels as >1.3 µg/mL

### Aggregate IgG responses

The percentages of aggregate responders were: subnormal IgG1, 54%; subnormal IgG3, 46%; and subnormal IgG1 + IgG3, 46%. In univariate analyses, the prevalence of atopy was significantly lower in patients who were aggregate IgG responders than in patients who were aggregate non-responders (Table 5). In a logistic multivariable regression on aggregate IgG responses, there were two significant associations: male sex (*p* = 0.03; OR 0.09 [95% CI 0.01, 0.77]); and atopy (*p* = 0.03; OR 0.17 [95% CI 0.03, 0.83]). Significance of the model was 0.083. This model accounted for 22.0% of the deviance of aggregate IgG responses.

**Table 5:** Characteristics of 59 index patients with subnormal IgGSc and total serum IgG > 700 mg/dL

Characteristic	Response^a^ (*n* = 31)	Non-response^a^ (*n* = 28)	Value of p^*^
Male, % (n)	9.7 (3)	25.0 (7)	0.1113
Mean age, y (± 1 SD)	47 ± 11	41 ± 13	0.3856
Mean body mass index, kg/m^2^ (± 1 SD)	27.7 ± 9.7	28.5 ± 6.4	0.7191
Autoimmune condition, % (n)	35.5 (11)	35.7 (10)	0.9853
Atopy, % (n)	12.9 (4)	46.4 (13)	0.0045
Other allergy, % (n)	4.8 (15)	53.6 (15)	0.6908
Subnormal IgG1 only, % (n)	19.4 (6)	25.0 (7)	0.6014
Subnormal IgG3 only, % (n)	51.6 (16)	60.7 (17)	0.4820
Subnormal IgG1 + IgG3 only, % (n)	29.0 (9)	14.3 (4)	0.1724
Median IgA, mg/dL	182	186	0.6132
Median IgM, mg/dL	110	89	0.7112

^a^Response was defined as protective levels of post-vaccination *S. pneumoniae* serotype-specific IgG (> 1.3 μg/mL) for ≥70% of serotypes tested after polyvalent pneumococcal polysaccharide vaccination

^*^Comparisons were made with Student’s unpaired t-test, Mann-Whitney U test, Pearson’s χ^2^ test or Fisher’s exact test, as appropriate. These are nominal values of *p*. Bonferroni correction for 11 comparisons yielded a revised *p* for significance of < 0.0045

## Discussion

The restriction of anti-pneumococcal polysaccharide IgG response post-PPSV23 was greater in our patients with subnormal IgG3 than in patients with subnormal IgG1. Previous studies have reported associations between subnormal IgG3 and respiratory tract infection [19, 27]. Risk for infections due to particular *S. pneumoniae* serotypes is probably increased in some patients with subnormal IgGSc due to their inability to synthesize specific protective antibodies. For example, impaired production of IgG anti-pneumococcal serotype 3 has been associated with recurrent pneumonia, empyema, septic shock, and increased fatality [28–30]. There is a positive association between invasive pneumococcal infection and impaired production of anti-serotype 4, 14, 51 and 56 [29]. IgG3 is also a key determinant in response to *Moraxella catarrhalis* and *S. pyogenes* infections [31, 32].

IgG3 antibodies in commercial intravenous IgG preparations have been reported to have the most potent binding and opsonic activity for *S. pneumoniae* serotype 6B [33], a major etiology of infections in persons with primary immune deficiency [34]. Individuals with subnormal serum IgA or heavy chain constant region deletions of the γ2 gene produce IgG1 and IgG3 anti-pneumococcal polysaccharide antibodies [35, 36]. Some patients with recurrent bacterial respiratory tract infections and subnormal IgG3 have subnormal subsets of blood T- or B-lymphocytes or impaired lymphocyte function [18, 23, 37, 38]. Patients with recurrent bacterial respiratory tract infections and subnormal IgG3 had less frequent or severe infections after they were treated with IgG devoid of IgG3 [39], suggesting that qualitative deficits in IgG1, IgG2, or possibly IgG4 subclasses increase infection susceptibility. Taken together, these observations indicate that multiple factors in individuals with subnormal IgG3, including suboptimal response to some *S. pneumoniae* serotype-specific antigens, contribute to increased susceptibility to respiratory tract infection.

In this study, patients with subnormal IgG3 and subnormal IgG1 + IgG3, PPSV23 responders had similar serum concentrations of anti-pneumococcal polysaccharide antibodies. In PPSV23 non-responders, the concentrations were higher in patients with subnormal IgG1 + IgG3. The greater responses to serotypes 8 and 9 and greater serum levels of IgG2 in patients with subnormal IgG1 + IgG3 could partly account for this difference.

In the cohort of 59 patients with subnormal IgGSc, there was a predominance of women, consistent with other subnormal IgGSc case series. A predominance of women was also observed in each of the three subnormal IgGSc immunophenotype subgroups we studied. In univariate analyses, we observed a strong positive association between low concentration of 1, 3, 14 and 51 serotype-specific antibodies and male gender. In a logistic regression on aggregate IgG responses, the odds of responses in men were low (0.09 [95% CI: 0.01, 0.77]). In an analysis of data extracted from 84 studies, Falagas and colleagues concluded that males in all age groups develop respiratory tract infections more frequently than females, except in cases of sinusitis, otitis externa, and probably tonsillitis [40].

There was an inverse correlation of PPSV23 response with age in the present study. This was most evident in non-responders with subnormal IgG1 (*r* = − 0.79) and responders with subnormal IgG3 (*r* = 0.71). In another study, IgG, IgA, and IgM concentrations were lower in elderly adults (aged 70–79 years) than in younger adults (college students) [41]. In another report, IgG responses to PPSV23 were stable in Prevnar13®-naive healthy adults as long as 6 years post-PPSV23 [42]. In the current study, the serum levels of post-PPSV23 *S. pneumoniae* serotype-specific IgG decreased with age, suggesting that the PPSV23 response stability of adults with subnormal IgGSc may be less durable than that of adults not selected for having subnormal IgGSc.

Univariate analyses demonstrated that restricted response to certain *S. pneumoniae* polysaccharide antigens was associated with increased prevalence of atopy (serotypes 4, 14, 56, and 68), other allergies (serotypes 3 and 51), and autoimmune condition(s) (serotypes 3 and 51) and with a predominance of male gender in patients with subnormal IgG3. In a regression analysis of aggregate response to PPSV23, atopy was associated with lower odds for responses, after adjustment for other variables. Atopy, defined as allergic asthma, allergic rhinitis, or allergic dermatitis/eczema, occurred in 29% of the present patients and in 36% of present patients with subnormal IgG3. Atopy and asthma were common in two case series of patients with subnormal IgG1 [7, 8] and in 121 adult patients with subnormal IgG3 [19]. Allergic asthma increases risk of lower respiratory tract infections in subjects not selected for subnormal immunoglobulin level [43, 44]. In another study, viral or bacterial infections were detected in 70% of inpatients with exacerbation of asthma [45]. Likewise, allergic rhinitis increases risk of sinusitis [46]. These observations suggest that the combination of restricted response to certain *S. pneumoniae* polysaccharide antigens and atopy increase the risk of respiratory tract infection in persons with subnormal IgGSc.

In the present adults, PPSV23 response rates decreased with increasing age. IgG responses of elderly adults to PPSV14 or PPSV23 measured by avidity were similar to those of younger adults [47, 48]. In contrast, concentration ratios of IgG1/IgG2 produced in response to an investigational 5-valent pneumococcal vaccine declined in adults with greater age for all serotypes studied (6B, 14, 18C, 19F, and 23F) [49]. In another study of elderly adults, post-vaccination IgG antibody concentrations for two serotypes (6B and 19F) of the five studied (4, 6B, 14, 19F, and 23F) were significantly lower and in vitro opsonophagocytosis of all five IgG serotype-specific antibodies was significantly reduced [50]. In two other reports, serotype-specific pre- and post-immune antibody responses measured by opsonophagocytosis were also significantly lower in elderly adults [51, 52]. IgG responses to PPS4 and PPS14 in elderly adults were associated with decreased V_H_ repertoire (mostly V_H_3) and somatic mutations and with greater loss of oligoclonality than younger adults [51]. Antigen responses may differentially involve more V_H_4 alleles in elderly than younger adults [53].

Aggregate IgG response was lower in the present men than women with IgGSc deficiency, after adjustment for other variables. Women have higher basal levels of plasma or serum IgG [54, 55]. Mean serum IgG levels in 26 men with Klinefelter’s syndrome were significantly higher than those of control men, but after androgen replacement therapy declined to mean levels similar to those of controls [56]. IgG responses to major pneumococcal serotypes in both pre- and post-PPSV23 sera were higher in elderly men than elderly women [48, 57, 58] but decreased significantly in women with increasing age [57]. Neither age, sex, IgG level, or IgG subclass levels were significant independent predictors of 4-fold or greater antibody response to PPSV23 [59].

The present results demonstrate that IgGSc responses to PPVS23 in adults with IgGSc deficiency are preferential and significantly associated with pre-vaccination IgGSc immunophenotypes. In IgG concentrates used to treat patients with primary antibody deficiency, IgG2 was the most active subclass of anti-*Streptococcus pneumoniae* antibodies by avidity and opsonophagocytosis measures, except that IgG3 was the most active against serotype 6B [33]. Relatively low responsiveness to PPSV23 of the present patients with subnormal IgG3 may be due in part to their inheritance of Gm3 polymorphisms (allotypes) [60, 61]. Two of the present patients had IgA deficiency. In another study, adults with IgG2/IgA deficiency had no IgG response to PPSV14 In normal adults, the predominant PPSV14 response occurred in IgG2 subclass [10], although PPSV14 responses also occur in other IgG subclasses [62]. In a subject with pneumococcal polysaccharide vaccination-specific IgG2 deficiency, the balance of specific IgG subclass antibodies changed from IgG2 > IgG1 > IgG3 > IgG4 pre-vaccination to IgG1 > IgG3 > IgG2 > IgG4 post-vaccination [6]. In patients with IgG2 deficiency, enhancement of IgG1 and IgG3 antibody responses to pneumococcal polysaccharide antigens suggest a change in normal switching or alteration in the antigen affinity of IgG subclass-specific antibody [36].

A strength of the present study is our evaluation of a sufficient number of adults with common subnormal IgGSc immunophenotypes to detect significant differences in some antigen-specific and aggregate IgG responses to PPSV23 and statistical associations of responses and non-responses to other variables. Limitations of the present study include exclusion of patients with subnormal IgG2 and lack of long-term follow-up of PPSV23 response stability. Investigating mechanisms by which variables such as male sex, atopy, and autoimmunity influence PPSV23 responses in patients with subnormal IgGSc was beyond the scope of the present study.

## Data Availability

All summary and many individual data generated or analysed during this study are included in this published article. Patient-by-patient datasets compiled and/or analysed during the current study are not publicly available because they could reveal personal identities.

## Abbreviations

ANOVA: Analysis of variance
BMI: Body mass index
CI: Confidence interval
Ig: Immunoglobulin
IgGSc: IgG subclass
OR: Odds ratio
PPS: Pneumococcal polysaccharide serotypes
PPSV23: Polyvalent pneumococcal polysaccharide vaccination with Pneumovax®23
SD: Standard deviation
